# Prevalence of chronic hepatitis C infection in the general population: results from a national survey, Estonia, July to December 2022

**DOI:** 10.2807/1560-7917.ES.2024.29.30.2300651

**Published:** 2024-07-25

**Authors:** Mira Hleyhel, Julia Geller, Amal Sadou, Paul Naaber, Tatiana Kuznetsova, Sigrid Vorobjov, Marleen Lõhmus, Martina Furegato, Suzanne Reed, Benjamin Bluemel, Erika Duffell, Kristi Rüütel

**Affiliations:** 1 Cerner Enviza/Oracle Life Sciences, Paris, France; 2 National Institute for Health Development, Tallinn, Estonia; 3 SYNLAB Estonia, Tallinn, Estonia; 4 Institute of Biomedicine and Translational Medicine, University of Tartu, Tartu, Estonia; 5 European Centre for Disease Prevention and Control (ECDC), Stockholm, Sweden

**Keywords:** Hepatitis C, prevalence, general population, chronic infection, seroprevalence

## Abstract

**Introduction:**

Obtaining epidemiological data on chronic hepatitis C virus (HCV) infection is essential to monitor progress towards the hepatitis C elimination targets.

**Aim:**

We aimed to estimate the prevalence of chronic HCV and the seroprevalence of HCV in the adult general population in Estonia.

**Methods:**

This cross-sectional study, conducted between 12 July and 6 December 2022, included anonymised residual sera collected prospectively from patients 18 years and older visiting a general practitioner in all counties of Estonia. Specimens were considered HCV-seropositive if they tested positive for HCV antibodies by enzyme-linked immunoassay, confirmed by line-immunoblot assay. Chronic HCV infection was determined by positive RT-qPCR.

**Results:**

We tested a total of 4,217 specimens. The estimated HCV seroprevalence and prevalence of chronic HCV infection were 1.8% (95% CI: 1.4–2.2) and 0.8% (95% CI: 0.5–1.1), respectively, with ca 8,100 persons estimated to have chronic HCV infection in the general adult population of Estonia. No statistically significant differences in the prevalence of chronic HCV infection were observed between sexes, counties or age groups, with the highest prevalence rates observed in men (sex ratio: 1.7), Ida-Virumaa County (1.8%; 95% CI: 0.8–3.6) and the age group 40–49 years (1.7%; 95% CI: 0.9–2.9).

**Conclusion:**

This study found an overall low prevalence of chronic HCV infection in Estonia. Continued efforts should be made for the targeted screening, diagnosis and treatment of individuals with chronic HCV infection to achieve hepatitis elimination targets.

**Key public health message**

**What did you want to address in this study and why?**

There were no recent national prevalence estimates for chronic hepatitis C virus (HCV) infection in Estonia. In the context of global and European targets to end viral hepatitis as a public health concern, estimating the hepatitis C prevalence is essential for guiding public health policies.

**What have we learnt from this study?**

Between 12 July and 6 December 2022, blood serum samples from 4,217 adults who visited general practitioners in all counties of Estonia were tested for HCV infection. Overall, the prevalence of chronic HCV infection was found to be low. Findings suggested higher rates among individuals aged 40–49 years, males and residents of Ida-Virumaa County.

**What are the implications of your findings for public health?**

The study provided an estimate of the current burden of HCV infection in Estonia and valuable epidemiological data that can inform the effective tailoring of hepatitis C prevention, case finding and treatment strategies.

## Introduction

Infection with hepatitis C virus (HCV) is generally asymptomatic or is associated with non-specific symptoms, and often remains undiagnosed for many years [1,2]. Around 70% of acutely infected individuals will develop persistent chronic infection [2], and of those who have the chronic form, some may develop chronic liver disease, cirrhosis and hepatocellular carcinoma [1,2]. Currently, HCV presents a significant global health burden with, according to the World Health Organization (WHO), ca 58 million people diagnosed with chronic HCV worldwide and ca 1.5 million new infections occurring every year [2]. The WHO’s 2016 Global Health Sector Strategy on Viral Hepatitis has set a target to end the viral hepatitis epidemic as a public health threat by 2030 [3]. In the past, HCV infection used to be treated with interferon (IFN)-based therapies that were associated with suboptimal outcomes and unfavourable safety profiles. However, the advent of the direct-acting antivirals (DAAs) led to a revolutionary improvement in the management of HCV infection. With their high efficacy and safety profile, these new agents make elimination of hepatitis C appear attainable [4,5]. One of the key elements for monitoring the progress towards the targets is the availability of HCV prevalence data, which are needed to estimate the number of individuals who need to be treated and thus guide the scaling up of prevention, testing and treatment of hepatitis C.

According to a recent meta-analysis involving 98 studies from around the world, the global prevalence of HCV in the general population was estimated at 1.8% (95% confidence interval (CI): 1.4–2.3) [6]. In Europe, studies conducted between 2000 and 2008 showed an estimated HCV prevalence ranging between ≤ 0.5% in northern European countries and ≥ 3% in Romania and rural areas in Greece, Italy and Russia [7]. 

In Estonia, a small country in northern Europe with a population of 1.3 million in 2023 [8], data on HCV prevalence in the general population are scarce. A single prevalence study was conducted in Estonia in 2018 (personal communication: Kristi Rusin, July 2023), which involved 503 consecutive patients (270 male and 233 female) aged 21–52 years in a general practice in the urban area of Tartu, the second largest city in Estonia. The data from this study showed that 1.8% (9/503) of the participants had HCV antibodies, with males being twice as likely as females to test positive. The mean age of anti-HCV-positive patients was 39.1 years overall, 42.5 years in male and 32.3 years in female participants. Furthermore, HCV RNA (indicative of a current HCV infection) was detected in 0.8% (4/503) of the participants. 

In the absence of a national prevalence survey for hepatitis C among the general population in Estonia, data from expert assessments and individual surveys may give an indication for the prevalence of HCV. The prevalence of chronic HCV infection in Estonia was estimated at 1.5% (95% CI: 1.1–1.9) in 2013 based on expert consensus, i.e. ca 20,000 individuals chronically infected with HCV [9]. A study by Mansberg et al. included patients with acute and chronic HCV infection as well as HCV-related cirrhosis and hepatocellular carcinoma who visited a gastroenterologist or an infectiologist between 2009 and 2010 in Estonia. This study reported that the main risk factors for HCV infection in Estonia were injecting drug use (IDU) and tattooing in the age group 30–49 years, and blood transfusion in the age group 50–69 years [10]. Genotypes 1 and 3 were the most common HCV genotypes [10].

Considering the absence of recent and representative national prevalence estimates for the general population, the objective of this cross-sectional study was to estimate the prevalence of chronic HCV infection and the seroprevalence of HCV in the adult population in Estonia.

## Methods

The methodology used in this study was based on the Technical Protocol for Hepatitis C Prevalence Surveys in the General Population – SPHERE C Project developed by the European Centre for Disease Prevention and Control (ECDC) [11].

### Study design and population

The target population of this survey was the adult population of Estonia. The study design involved the prospective collection of anonymised residual sera from patients aged 18 years or older visiting a general practitioner (GP) between 12 July and 6 December 2022, from whom a venous blood sample was collected by a GP for routine medical evaluations, examinations conducted as part of preventative medical care and various diagnostic purposes. Specimens from patients who visited a GP were included in this study, as this is a primary care setting where a multitude of health problems are treated and where patients without previous complaints also come for a check-up. Therefore, to prevent an over-representation of individuals with a potentially higher HCV prevalence than that of the general population, specimens from sources other than GPs were excluded for this study, such as those collected from drug treatment centres, sexually transmitted infection (STI) clinics, or from hepatology or infectious disease services. Specimens specifically submitted for HCV screening were also excluded.

Specimens collected by the GPs were then sent to a medical laboratory (SYNLAB Estonia) for analysis. The SYNLAB Estonia provides laboratory services to approximately three quarters of GPs all over Estonia [12].

### Sample size

Taking into consideration the existing data and the expected reduction of HCV prevalence over time in the general population (through individuals who were cured with treatment or who died), the expected prevalence of chronic HCV infection was set at 1% for the sample size calculation. With an expected prevalence of 1%, a desired precision of 0.3%, a confidence level of 95% and a design effect of 1, a total minimum sample size of 4,226 was estimated. The sample in each age group, sex and county stratum was calculated according to the probability proportional to the size of the corresponding strata in the general population in Estonia. The regional groupings considered the size of the counties: Harjumaa, Tartumaa, Ida-Virumaa, Pärnumaa and ‘Other’, the latter including the remaining 11 counties due to their small population size (Hiiumaa, Järvamaa, Jõgevamaa, Läänemaa, Lääne-Virumaa, Põlvamaa, Raplamaa, Saaremaa, Valgamaa, Viljandimaa, Võrumaa).

Based on the calculated sample size in each age group, sex and county stratum, the precision for the 95% CI of the estimated HCV prevalence was expected to be 0.4% in the sex strata, 0.7–0.8% in the age strata and 0.4–1.2% in the county strata.

### Sampling procedure

We selected the specimens using the pre-defined inclusion and exclusion criteria by age, sex and county from ca 300,000 samples collected by GPs in the period from July to December 2022. Selection of the specimens from the sera submitted by GPs to the laboratory was automatised using the laboratory information system (LIS) and was done weekly during four working days (Tuesday through Friday). Samples were systematically identified and retrieved from the archive until the required number was achieved. In addition, we included specimens in the study if at least 48 h had elapsed since sample collection and if specimen volume was greater than 500 µL. Within the age group, sex and county strata, we selected specimens by simple random sampling until the required number was reached.

### Laboratory testing algorithm and interpretation of results

Laboratory testing was performed at the National Institute for Health Development. Initial screening for anti-HCV antibodies in all specimens was performed using the semi-automated Murex anti-HCV enzyme-linked immunosorbent assay (ELISA) (version 4.0) (DiaSorin, South Africa Ltd; REF 7F51/06/-07). Anti-HCV-reactive specimens were investigated for HCV RNA by the GeneProof HCV Diagnostic PCR Kit. Finally, the anti-HCV line-immunoblot assay (LIA) recomLine HCV IgG (Mikrogen Diagnostics) tests were used to confirm the anti-HCV results in HCV RNA-negative specimens.

Specimens were considered as HCV-seropositive (chronic or resolved infection) if they tested positive for HCV RNA in RT-qPCR or positive for HCV antibodies by ELISA which was confirmed by positive LIA. Specimens that tested positive for HCV in RT-qPCR were considered indicative of chronic HCV infection, while those that were RT-qPCR-negative but positive in the immunoblot assay were classified as resolved HCV infections. Specimens with an undetermined result for the LIA and negative for HCV RNA in RT-qPCR were classified as having an undetermined HCV status. Samples that did not meet any of these criteria were classified as HCV-seronegative.

### Statistical analysis

The seroprevalence (either resolved or chronic infections) and the prevalence of chronic HCV infection and their 95% CI were calculated overall and by age group, sex and county. We used chi-squared tests to compare the prevalence of chronic HCV infection and the seroprevalence between the two sexes, age groups and counties. To gain further insights, we conducted pair-wise p value comparisons, which involved systematically comparing combinations of each age group pair and each county pair. This analysis allowed us to investigate whether there were any notable differences in the prevalence among different age groups or counties. Bonferroni multiple significance test correction was applied. Finally, we estimated the number of persons seropositive or chronically HCV infected in the adult population in Estonia overall and by age group, sex and county by applying the prevalence estimations in the sample to the Estonian population data of 2021 [13]. SAS version 9.4 was used for the statistical analyses.

## Results

A total of 4,217 specimens were collected and tested for HCV infection. The demographic characteristics of the sample are presented in Table 1. The survey sample was representative of the general population in Estonia in terms of sex, age and geographic distribution. Male participants accounted for 46.4% of the total sample, with the sex distribution relatively balanced across the six age groups. Slightly less than half of the sample (45.5%) came from Harjumaa county.

**Table 1:** Demographic characteristics of the study sample, Estonia, July–December 2022 (n = 4,217), and the general adult population in 2021

Demographic characteristics	Study population n = 4,217		Total Estonia population [13] n = 1,069,293	
	n	%	n	%
Sex				
Male	1,956	46.4	496,683	46.4
Female	2,261	53.6	572,610	53.6
Age group (years)				
18–29	642	15.2	163,405	15.3
30–39	783	18.6	197,604	18.5
40–49	717	17.0	182,143	17.0
50–59	673	16.0	170,485	15.9
60–69	647	15.3	164,421	15.4
≥ 70	755	17.9	191,235	17.9
County				
Harjumaa	1,918	45.5	486,028	45.5
Ida-Virumaa	437	10.4	110,534	10.3
Pärnumaa	274	6.5	69,303	6.5
Tartumaa	474	11.2	120,139	11.2
Other	1,114	26.4	283,289	26.5

Among the 4,217 specimens, 101 (2.4%) were found anti-HCV ELISA-positive and underwent subsequent RT-qPCR testing. Of these 101 specimens, 32 (31.7%) were considered as having chronic HCV infection, while 69 (68.3%) tested negative for the presence of HCV RNA in RT-qPCR and underwent LIA testing. The LIA results showed that of the 69 specimens, 42 (60.9%) were seropositive with resolved infection, 18 (26.1%) were seronegative, and nine (13.0%) were undetermined in terms of their HCV status (Figure).

**Figure:**
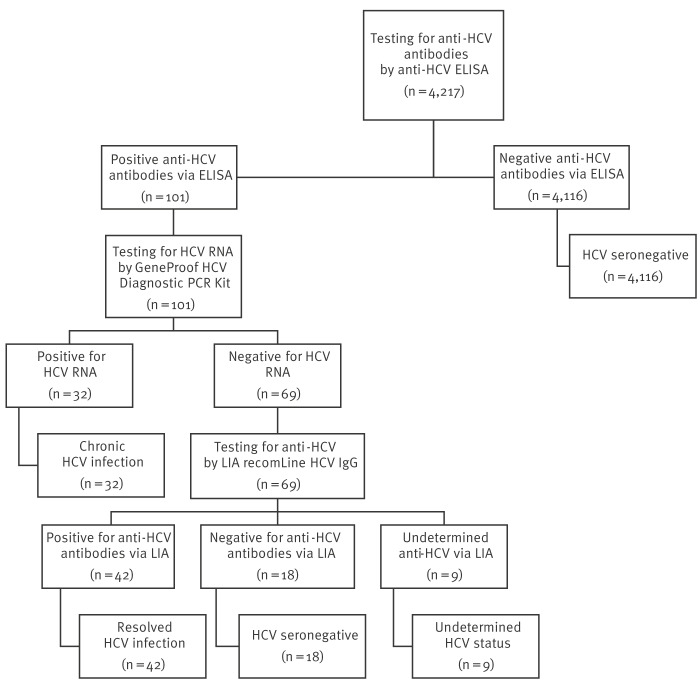
Hepatitis C virus testing algorithm and interpretation of test results, Estonia, July to December 2022 (n = 4,217)

Ab: antibodies; Ag: antigen; ELISA: enzyme-linked immunosorbent assay; HCV: hepatitis C virus; LIA: line immunoblot assay.

### Seroprevalence

Of the 4,217 specimens, 74 were confirmed seropositive, giving a prevalence of 1.8% (95% CI: 1.4–2.2) (Table 2). Based on this, we estimated that 18,750 individuals in the adult general population in Estonia are or were previously infected with HCV.

**Table 2:** Estimated hepatitis C seroprevalence and estimated number of seropositive individuals in the general adult population, Estonia, 2022 (n = 4,217)

Demographic characteristics	Number of specimens in the study sample	Number of HCV seropositive specimens in the study sample	HCV seroprevalence in study population		Estimated number of HCV seropositive individuals in Estonia^a^	
			%	95% CI	n	95% CI
All	4,217	74	1.8	1.4–2.2	18,754	14,519–22,989
Sex						
Female	2,261	27	1.2	0.8–1.7	6,838	4,274–9,402
Male	1,956	47	2.4	1.8–3.2	11,941	8,568–15,313
Age groups (years)						
18–29	642	1	0.2	0.0–0.9	255	0–753
30–39	783	17	2.2	1.3–3.5	4,290	2,273–6,307
40–49	717	23	3.2	2.0–4.8	5,843	3,494–8,192
50–59	673	14	2.1	1.1–3.5	3,546	1,708–5,385
60–69	647	9	1.4	0.6–2.6	2,287	803–3,771
≥ 70	755	10	1.3	0.6–2.4	2,533	973–4,092
County						
Harjumaa	1,918	36	1.9	1.3–2.6	9,123	6,171–12,074
Ida-Virumaa	437	17	3.9	2.3–6.2	4,300	2,296–6,304
Pärnumaa	274	4	1.5	0.4–3.7	1,012	27–1,996
Tartumaa	474	7	1.5	0.6–3.0	1,774	470–3,079
Other	1,114	10	0.9	0.4–1.6	2,543	974–4,112

CI: confidence interval; HCV: hepatitis C virus.

^a^ The number of seropositive individuals in Estonia was calculated based on the prevalence estimates and the Estonian population data of 2021 [13].

The HCV seroprevalence was significantly higher in males (2.4%; 95% CI: 1.8–3.2%) compared with females (1.2%; 95% CI: 0.8–1.7) and higher in specimens from Ida-Virumaa County (3.9%; 95% CI: 2.3–6.2) compared with other counties with small populations combined (0.9%; 95% CI: 0.4–1.6).

Age group comparisons showed that the seroprevalence was significantly higher in the age groups 30–39 years (2.2%; 95% CI: 1.3–3.5), 40–49 years (3.2%; 95% CI: 2.0–4.8) and 50–59 years (2.1%; 95% CI: 1.1–3.5) compared with the 18–29-year-olds (0.2%; 95% CI: 0.0–0.9). We found only one case of confirmed seropositivity in the latter age group.

### Prevalence of chronic hepatitis C virus infection

A total of 32 specimens were confirmed as having chronic HCV infection, indicating a prevalence of chronic infection of 0.8% (95% CI: 0.5–1.1), with a male-to-female sex ratio of 1.7 (Table 3). Based on this prevalence rate, it was estimated that there were ca 8,100 persons with chronic HCV infection in the general population in Estonia.

**Table 3:** Estimated prevalence of chronic hepatitis C virus infection and estimated number of individuals with chronic hepatitis C virus infection in the adult general population, Estonia, 2022 (n = 4,217)

Demographic characteristics	Number of specimens in the study sample	Number of specimens with chronic HCV infection in study sample	Prevalence of chronic HCV infection in study population		Estimated number of individuals with chronic HCV infection in Estonia^a^	
			%	95% CI	n	95% CI
All	4,217	32	0.8	0.5–1.1	8,117	5,315–10,919
Sex						
Female	2,261	13	0.6	0.3–1.0	3,292	1,508–5,077
Male	1,956	19	1.0	0.6–1.5	4,827	2,667–6,987
Age groups (years)						
18–29	642	1	0.2	0.0–0.9	255	0–753
30–39	783	4	0.5	0.1–1.3	1,009	23–1,996
40–49	717	12	1.7	0.9–2.9	3,048	1,338–4,759
50–59	673	5	0.7	0.2–1.7	1,267	161–2,373
60–69	647	6	0.9	0.3–2.0	1,525	310–2,739
≥ 70	755	4	0.5	0.1–1.4	1,013	23–2,003
County						
Harjumaa	1,918	15	0.8	0.4–1.3	3,801	1,885–5,717
Ida-Virumaa	437	8	1.8	0.8–3.6	2,024	634–3,413
Pärnumaa	274	3	1.1	0.2–3.2	759	0–1,613
Tartumaa	474	0	0.0	NA	0	NA
Other	1,114	6	0.5	0.2–1.2	1,526	308–2,743

CI: confidence interval; HCV: hepatitis C virus; NA not applicable.

^a^ The number of chronically infected individuals in Estonia was calculated based on the prevalence estimates and the Estonian population data of 2021 [13].

Although not statistically significant, there was a higher prevalence of chronic HCV infection among specimens from Ida-Virumaa County (1.8%; 95% CI: 0.8–3.6) than among samples from other counties. No chronic infections were found in Tartumaa.

There were no statistically significant differences in the prevalence of chronic HCV infection between age groups, with the highest prevalence in the 40–49-year-olds (1.7%; 95% CI: 0.9–2.9) and lowest in the 18–29-year-olds (0.2%; 95% CI: 0.0–0.9) (p = 0.062).

## Discussion

This study of HCV prevalence in the general population in Estonia suggests that Estonia is a country with a low endemicity for chronic HCV. Our results also indicate that men have the highest prevalence of chronic HCV infection, with a male-to-female sex ratio of 1.7. In addition, our results suggest a higher prevalence of chronic HCV infection in Ida-Virumaa County and among individuals aged 40–49 years.

The prevalence rates of HCV infection found in this study were similar to a previous study conducted in 2018 in Tartu, a county in Estonia, which detected HCV antibodies and HCV RNA in 1.8% and 0.8% of participants, respectively (personal communication: Kristi Rusin, July 2023). The prevalence estimates in the current study (1.8% for HCV seroprevalence and 0.8% for the prevalence of chronic HCV infection) were lower than the prevalence of chronic HCV infection of 1.5–2% estimated for the entire country by the Estonian Society of Gastroenterologists and the Estonian Society of Infectious Diseases in 2013 [9]. Despite methodological differences between studies in the generation of estimates which could account for changes in prevalence over time, we can speculate that the reduction in HCV prevalence from the 2013 estimate may be real. This reduction may be partially explained by the death of individuals with chronic HCV from older cohorts [14] as well as the cure of some infections due to the major advancements in treatment in recent years with the emergence and roll-out of the highly effective DAAs. In addition, some reports suggest that the size of the population of people who inject drugs (PWID) in Estonia is decreasing, and in particular that fewer people are initiating injecting [15,16], which may lead to a decrease in the incidence of hepatitis C infection in this population, as IDU used to be one of the main transmission routes for HCV infection in Estonia. Between 2010 and 2015, it was estimated that the number of PWID was 6,000–17,300, with a slight decrease observed over these 5 years [16]. Of note, up to 80% of HIV-positive PWID are also co-infected with HCV [17].

The estimated prevalence of chronic HCV infection in Estonia in our study was lower than estimates of prevalence from neighbouring countries such as Latvia. The most recent general population study in Latvia was conducted in 2008, used a robust sampling strategy (multistage randomised selection) among primary care physicians in different regions of the country, and resulted in a prevalence of 2.4% (95% CI: 1.7–3.3) [18]. However, it may be expected that the current prevalence of HCV infection in Latvia is lower than the 2008 estimate on account of the efforts to scale up testing, including actively inviting people for testing by healthcare providers, and treatment that have taken place in relation to the elimination agenda.

Our results also show that men are more likely than women to have been infected with HCV, likely due to increased frequency of exposure. Indeed, the seroprevalence of HCV in men (estimated at 2.4%) was twice that of women (seroprevalence of 1.2%). Our findings are consistent with the results of previous HCV prevalence studies in other countries [18,19] and with the findings of an ECDC report showing that men had a higher notification rate of newly diagnosed hepatitis C across Europe [20]. Similar to other countries [21], this may be explained by the fact that IDU is the main risk factor for HCV infection in Estonia [10,16] and men are more likely than women to engage in risky behaviours such as injecting drugs [22] and sharing contaminated syringes [23].

We found that the seroprevalence was highest in individuals aged 40–49 years (3.2%), followed by those aged 30–39 years (2.2%). These findings are similar to those of Mansberg et al. [10], and probably reflect the higher HCV burden in these age groups due to historic trends in IDU in Estonia. Injecting drug use started increasing in the country from 1995 onwards, particularly among young males between the ages of 15 and 24 years [22,24,25], before eventually decreasing from 2.7% (95% CI: 1.8–7.9) in 2005 to 0.9% (95% CI: 0.7–1.7) in a population aged 15–44 years in 2009 [15]. It is therefore reasonable to speculate that individuals who are currently between 40 and 49-years-old correspond to those who were most affected by the beginning of the drug use epidemic in Estonia. A high seroprevalence and prevalence of chronic HCV infection was also observed in the age group 50 years and older who may have acquired HCV from blood transfusion before 1994 [10].

Our survey showed higher seroprevalence and prevalence of chronic HCV infection in Ida-Virumaa County compared with the other counties. This finding is consistent with regional differences in IDU as a public health issue in Estonia. The majority of PWID are concentrated in two specific areas, namely the capital city of Tallinn in Harjumaa County and in Ida-Virumaa County [15,26]. Although the number of people with chronic HCV infection was notably higher in Harjumaa County (n = 15) than in Ida-Virumaa County (n = 8), the prevalence of chronic HCV infection was lower in Harjumaa County (0.8%; 95% CI: 0.4–1.3), the largest county in Estonia in terms of population, than in Ida-Virumaa County (1.8%; 95% CI: 0.8–3.6) which has a smaller population. Although local treatment data were not available, it is likely that these differences were related to HCV treatment being more readily available in the more populous county, which may explain the lower prevalence in Harjumaa County.

We estimate that 10,600 individuals had resolved HCV infection in Estonia. We consider this a reasonable estimate given that the cumulative number of HCV-infected patients who received treatment between 2005 and 2018 was ca 6,500 in Estonia [27] (rising to 8,000 patients by 2022, assuming estimates of annually treated patients remained stable in subsequent years) and that the spontaneous clearance rate for acute HCV infection was estimated at 25% [28]. Unfortunately, no data are available on the risk group distribution of people treated for HCV and how many of those treated were actually cured. There is no national registry of HCV treatment, and thus the 8,000 patients treated by 2022 are only an estimate based on national health insurance data. It is speculated that campaigns to increase awareness of HCV and promote testing have resulted in a continued uptake of treatment, but these efforts cannot be quantified with currently available data.

One of the main strengths of our study is that it is a national survey representative of the adult general population in Estonia regarding age, sex and geographic area. The sample size was close to the calculated minimum sample size, and this ensured sufficient precision in both overall estimates of a general population and specific subgroups of interest. In addition, the study used specimens that had already been collected for other diagnostic and screening investigations. This facilitated the estimation of prevalence rates in a timely and cost-effective manner.

One limitation of our study is the potential for selection bias, as specimens collected from individuals attending GP clinics may not be fully representative of the broader adult population i.e. those not covered by the country’s national health insurance (ca 5% of the population in Estonia) [29], those younger and thus healthier, those who may fear stigma from GPs if they have a history of IDU, or individuals with no medical symptoms who do not attend GP clinics. In 2012, it was estimated that only slightly more than half of PWID in Estonia had health insurance coverage, which would be a barrier to healthcare access, be it a visit to the doctor or undergoing laboratory testing [15]. However, our specimens were more representative than specimens collected in specialist clinics which are more likely to be attended by individuals with more serious health conditions and who may have a higher prevalence of HCV infection than those visiting GPs. Other studies have used a similar design for HCV prevalence estimations, and this methodological approach is considered to result in valid prevalence rates [19]. To minimise the selection bias, we excluded certain categories of residual specimens such as those submitted specifically for HCV testing. The aim of these exclusions was to prevent an over-representation of individuals from high-risk groups, which could lead to an overestimation of the prevalence estimations. A further limitation was that the design of the study using anonymised residual sera did not allow collecting information on risk factors for hepatitis C infection.

## Conclusion

The prevalence of chronic HCV infection in the general population of Estonia is low. Conducting a prevalence survey to estimate chronic HCV infection in Estonia has been important to estimate the needs for DAA treatment at the national level and to support the development of national screening strategies. Conducting additional population-based surveys on a regular basis in the future is crucial for monitoring the progression of the epidemic over time and the effectiveness of preventive and control measures. Future research should include the collection of behavioural data to assess risk factors for HCV infection. Given the low level of resources needed to conduct this study, implementing the same design in future studies would serve as an effective method to monitor progress towards HCV elimination.
